# Small Intestinal Volvulus Caused by Lipomatosis in a Middle-Aged Female

**DOI:** 10.1155/2023/7944187

**Published:** 2023-09-07

**Authors:** Deepak Kumar, Shashikant Kumar, Anurag Kumar, Shreekant Bharti, Majid Anwer, Anil Kumar

**Affiliations:** ^1^Department of General Surgery, All India Institute of Medical Sciences, Patna 801507, India; ^2^Department of Trauma Surgery, All India Institute of Medical Sciences, Patna 801507, India; ^3^Department of Pathology/Laboratory Medicine, All India Institute of Medical Sciences, Patna 801507, India

## Abstract

Lipomas are benign tumors composed of adipose tissue that can occur in various locations throughout the body, including the gastrointestinal (GI) tract. Lipoma of the small bowel is a rare clinical condition. It infrequently results in small bowel obstruction and volvulus. In most of the patients, it is an incidental diagnosis. However, it may present with abdominal pain, nausea, vomiting, GI bleeding, and constipation. It is commonly diagnosed using imaging studies, such as computerized tomography scan or magnetic resonance imaging. The management of small bowel lipomas depends on the presence of symptoms and the risk of complications. Asymptomatic lipomas can be safely monitored with imaging studies, as the risk of complications is low. However, in symptomatic cases, surgical or endoscopic treatment may be necessary to relieve obstruction and prevent complications. We report a case of volvulus of small bowel in a middle-aged female presenting to our hospital with acute GI symptoms.

## 1. Introduction

The incidence of intestinal lipomas varies from 0.0035% to 4.44%. Small bowel lipomas are benign adipose growths, usually found within the bowel wall or mesentery of the small intestine, and are rarely encountered [1]. Intestinal lipoma of the small bowel in 90% of cases arises from the submucosa of the small bowel [2]. Small bowel lipomas are usually an incidental finding and most of the patients are usually asymptomatic. The most frequent site in the small intestine is the ileum (54%), followed by the duodenum (32%) and jejunum (14%) [3]. Histologically, intestinal lipomas are of three types: the submucosal type, the intramuscular type, and the intermuscular type. The sub-serosal types are the less common ones, but are responsible for small bowel volvulus [4]. These benign slow growing mesenchymal tumours may cause intussusception or ulceration or gastrointestinal (GI) bleeding and or iron deficiency anaemia.

## 2. Case Report

**Figure 1:**
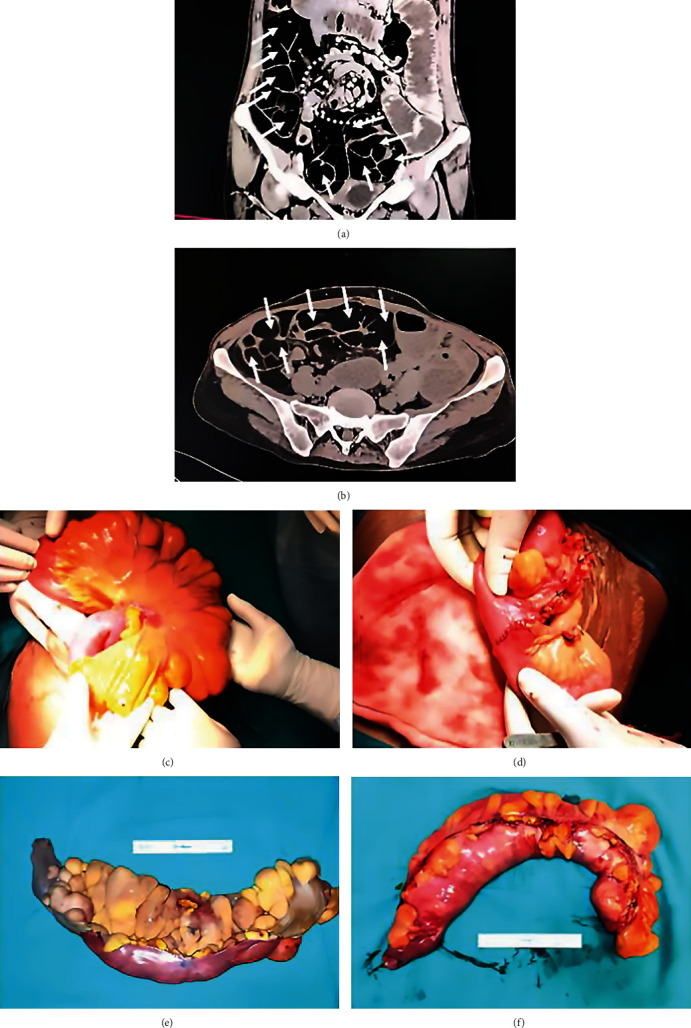
(a–f) CECT scan of the lower abdomen showing dilated bowel segments and fat-intense soft tissue lesions in the mesentery. White solid arrows indicating the small bowel lipomatosis (a and b) with the white interrupted circle (in a) showing rotated mesentery. Intraoperative findings of increased adipose tissue forming nodular masses along the mesentery (c, e, and f) and a volvulus segment relieved by detorsion and ileo-ileum anastomosis (d).

A 47-year-old female was admitted to the emergency department with complaints of abdominal pain and inability to pass stool for five days and nausea associated with vomiting of around eight to ten episodes per day. The patient was on treatment for dyspepsia and constipation for the last one month. The patient had a history of bilateral fallopian tube ligation fifteen years back. No history of any other co-morbidities was noted. On examination, the patient's pulse rate was 82 beats per minute, blood pressure of 108/49 mm Hg, and was maintaining saturation at room air. Per-abdomen examination revealed a soft, non-distended, non-tender abdomen without any palpable mass or, visible peristalsis. There was no organomegaly. Normal bowel sounds were heard. Her routine haematological and biochemical investigations were all within normal limits except for having mild anaemia (Hb 9.2 g/dl). Other systemic examinations did not reveal anything noticeable. Abdominal radiographs taken, however, showed multiple air fluid levels with dilated bowel loops suggestive of small bowel obstruction. Contrast-enhanced computed tomography (CECT) of the whole abdomen and pelvis was done. CECT revealed features likely of midgut volvulus resulting in intestinal obstruction with long segmental ileal lipomatosis (Figures 1(a) and 1(b)).

The patient was taken up for laparotomy. Intra-operative findings revealed dilated proximal bowel loops. A jejunal volvulus was noted. An approximate 45 cm length of distended ileal loop 30 cm proximal to ileocecal junction with ileal lipomatosis was noted. The mesenteric border showed relatively increased fatty deposition. There was no evidence of bowel ischemia or necrosis. The ileal segment of 45 cm containing lipomatous swellings was resected after detorsion of jejunal volvulus and hand-sewn double layer end-to--to-end ileo-ileal anastomosis was done (Figures 1(c), 1(d), and 1(e)). Minimal blood loss was noted (<100 m) not requiring any blood or blood product transfusion. At the end of the procedure, a 30-French drain was placed in the pelvic cavity. The patient's postoperative hospital stay was uneventful. The specimen was sent for a histological examination, which confirmed a benign lipomatous lesion. The patient was discharged on the tenth postoperative day. A follow-up sonograph after six months of discharge revealed no recurrent lesion. The patient did not report any complaints as well.

## 3. Discussion

Less than 150 occurrences of mesenteric lipomas have been documented in English literature, making them an uncommon clinical entity. Its etiopathogenetic pathway is still unclear. According to the available reports, there is a higher prevalence in individuals who are obese, have comorbidities like diabetes mellitus or hypercholesterolemia, have had trauma or radiation, have a family history of the condition, or have certain chromosomal abnormalities [5]. The patient in our situation lacked any of these risk factors. These lipomas seldom affect children under the age of ten years and are more frequent in adults between the ages of 40 and 60 years. Different types of symptoms might be brought on by lipomas depending on their size and location. They are asymptomatic and soft, movable masses that do not invade surrounding tissues, allowing the passage of intestinal material [6].

Farkas et al. in their systematic review of symptomatic small bowel lipoma found that abdominal pain was the most common presentation in 68.7% as was in our case, followed by nausea and vomiting in 35.3%. Other presentations may include hematochezia/GI bleeding (33%), abdominal distension (12.2%), constipation (8.9%), and weight loss (7.5%) [7].

Small intestinal lipomas are frequently discovered incidentally in asymptomatic people. An abdominal X-ray is useless for diagnosis. Although a mesenteric cyst may be distinguished from it by ultrasound thanks to its well-defined homogeneous echogenic mass, it is typically impacted by visceral fat or intestinal gas. Due to this, even though it is the examination of the first choice for diagnosing intestinal obstruction caused by volvulus in infants, it does not allow for the accurate definition of the tumour's boundaries or placement concerning the peritoneum [8]. The gold standard imaging technique for determining the presence of mesenteric lipoma and other lipomatous abdominal tumours is the CT scan [9–11]. It provides details on the small bowel's characteristics, a homogeneous tumour of adipose tissue, and whether any evidence is there for a typical “vortex” pattern of volvulus. CT scan allows differentiation of liposarcomas from benign lipomatous lesions. A well-differentiated liposarcoma can be differentiated from lipomatosis in having a usually large size of more than 10 cm, a well-defined globular mass with a low percentage of fat within, often displaying thicker intratumoral septae (usually >2 mm). Detection of atypical lipoblasts in histology along with special immunohistochemical markers substantiates the diagnosis. In contrast, lipomatosis is considered a hypodense and fat proliferative lesion with occasional thin intratumoral septae on CT scan.

As far as treatment is concerned, small lipomas are usually left untreated, however, there have been some reports of endoscopic mucosal resection of ileal lipomas [12], but in general treatment of mesenteric lipoma is mainly done by complete surgical resection sparing the loop of bowel if possible. As of now, there has been no consensus on the best treatment modality. Laparoscopic resection of small lipoma has been advocated by Watt et al. [13] and Tsushimi et al. [14]. However, Kakiuchi et al. [15] stated that an extended umbilical incision may be required in case of large-size lipoma. Often treatment of large intestinal lipoma requires surgical resection and anastomosis as done in this case [16].

## 4. Conclusion

We report a rare case of mid-gut volvulus caused by lipomatosis of the ileum. Lipoma of the small intestine, though relatively common, very rarely causes volvulus of the intestine. The consideration of this entity as a causative factor for intestinal volvulus may help in proper treatment and prognostication, as they can be successfully managed by surgical resection.

## Data Availability

The intraoperative clinical images and the radiological findings are already included in the manuscript.
